# Combined effects of apparent low temperature and PM_2.5_ pollution on circulatory emergency ambulance calls: a time-series analysis in Shijiazhuang, China

**DOI:** 10.3389/fpubh.2026.1838363

**Published:** 2026-07-07

**Authors:** Siyuan Chen, Mengna Li, Yishan Ding, Ning Xu, Mingyang Guan, Fengge Chen, Junwang Tong

**Affiliations:** 1 Department of Public Health Monitoring and Assessment, Shijiazhuang Center for Disease Control and Prevention, Shijiazhuang, China; 2 School of Public Health, North China University of Science and Technology, Tangshan, China; 3 School of Public Health, Hebei Medical University, Shijiazhuang, China; 4 Research Base for Environment and Health in Shijiazhuang, Chinese Center for Disease Control and Prevention, Shijiazhuang, China; 5 Hebei Key Laboratory of Intractable Pathogens, Shijiazhuang, China

**Keywords:** apparent temperature, circulatory system diseases, emergency ambulance calls, joint exposure, PM_2.5_, time-series analysis

## Abstract

**Background:**

The health impacts of compound environmental exposures, particularly the joint effects of low temperature and air pollution, remain insufficiently understood.

**Objective:**

This study aimed to evaluate the independent and joint effects of apparent low temperature (AT) and PM_2.5_ on cardiovascular emergency ambulance calls (EACs). Methods: Daily cardiovascular emergency ambulance calls (EACs), meteorological, and air pollution data in Shijiazhuang, China (2014–2023) were analyzed. AT was calculated using the Steadman formulation. Distributed lag non-linear models (DLNM) were applied to assess lagged effects, while joint exposure models and relative excess risk due to interaction (RERI) were used to evaluate joint effects and interactions.

**Results:**

A total of 82,714 EACs were included. Apparent low temperature was significantly associated with increased risk of cardiovascular EACs, with delayed effects. Most joint exposure scenarios further elevated risks, with the strongest cumulative effect observed under the P1T3 scenario (*RR* = 1.146, 95% CI: 1.088–1.207). Subgroup analyses showed higher susceptibility among males, older adults, and patients with hypertension. Both synergistic and antagonistic interactions were identified.

**Conclusion:**

Combined exposure to apparent low temperature and PM_2.5_ significantly increases the risk of cardiovascular emergency ambulance calls, with complex lagged and heterogeneous effects. These findings provide important epidemiological evidence for understanding compound environmental risks and underscore the need to incorporate multi-factor environmental exposures into public health early warning and intervention strategies.

## 1 Introduction

Climate change has become one of the most significant global public health challenges (1). A growing body of epidemiological evidence has demonstrated that exposure to non-optimal temperatures is significantly associated with increased mortality risk, with the health effects of low temperatures being particularly pronounced (2, 3). Previous studies have further shown that cold exposure is closely linked to increased risks of non-accidental mortality as well as cardiovascular and respiratory deaths (4).

Meanwhile, air pollution remains a major environmental health risk worldwide. The Global Burden of Disease study has highlighted the substantial health burden attributable to air pollution, among which fine particulate matter (PM_2.5_) is considered one of the leading causes of mortality, with especially significant impacts on circulatory system diseases (5, 6). Although air quality has improved in recent years, PM_2.5_ concentrations in developing countries such as China still frequently exceed the World Health Organization guideline levels, and the associated health risks remain considerable (7–9).

There are complex interactions between temperature variations and air pollution. PM_2.5_ concentrations typically exhibit strong seasonal patterns; during winter, unfavorable meteorological conditions and cold air transport can facilitate regional pollutant transmission and accumulation in downwind areas (10). After temporary dispersion by cold air masses, pollutant levels often rebound rapidly, leading to compound exposure scenarios characterized by “low temperature combined with high pollution” (11). With the intensification of climate change, such compound environmental events are likely to occur more frequently and may exert additive or even synergistic effects on human health (12, 13).

Despite increasing attention to climate-related health risks, most existing studies have focused on single environmental factors and have primarily used mortality or hospital admissions as health outcomes (14, 15). Evidence on the health impacts of combined exposure to low temperature and air pollution, particularly on acute health events, remains limited (16). In addition, apparent temperature (AT), which integrates air temperature, relative humidity, and wind speed, better reflects the actual thermal perception of the human body (17). However, the application of AT in epidemiological studies is still relatively limited, especially in the context of compound environmental exposures.

Shijiazhuang, located in the Beijing–Tianjin–Hebei region of northern China, has a typical temperate continental monsoon climate, characterized by cold and dry winters. Combined with urban heat island effects and regional air pollution, residents in this area are exposed to complex environmental health risks arising from the joint effects of temperature variation and air pollution. Based on ambulance dispatch data for circulatory system diseases, along with meteorological and air pollution data in Shijiazhuang from 2014 to 2023, this study applied a distributed lag non-linear model (DLNM) to systematically assess the impact of apparent low temperature on ambulance dispatches for circulatory system diseases. Furthermore, by incorporating high temporal-resolution emergency medical data as the health outcome and using apparent temperature to characterize cold exposure, this study explored the joint effects of low temperature and PM_2.5_ from the perspective of compound exposure. The findings of this study may provide new evidence for understanding the acute health impacts of compound environmental risks under climate change and offer scientific support for developing targeted public health warning systems and intervention strategies.

## 2 Materials and methods

### 2.1 Data on circulatory emergency ambulance calls (EACs)

Daily EACs data from 1 January 2014 to 31 December 2023 were obtained from the Shijiazhuang Emergency Medical Center. The dataset included demographic characteristics (age and sex), chief complaints, and clinical diagnoses, and comprehensively recorded all EACs initiated during the study period. To ensure data confidentiality, sensitive personal identifiers, such as patient names, residential addresses, and contact information, were encrypted prior to data extraction.

Daily EACs were aggregated according to the International Classification of Diseases, 10th Revision (ICD-10), including circulatory system diseases (I00–I99), cerebrovascular diseases (I60–I69), heart diseases (I20–I25, I05–I09, I26–I28, I30–I52), and hypertension (I10). ICD-10 classification was based on the primary prehospital clinical diagnosis recorded by emergency physicians at the time of ambulance dispatch and on-scene assessment, rather than hospital discharge diagnoses. To reduce potential outcome misclassification, all records were coded according to standardized emergency medical service protocols routinely used by the Shijiazhuang Emergency Medical Center.

Air pollution and meteorological data were obtained from the Shijiazhuang Environmental Forecasting Center and the Shijiazhuang Meteorological Service Center, respectively. These data mainly included daily mean concentrations of PM_2.5_ and O_3_, as well as daily mean temperature (T, °C), relative humidity (RH, %), and wind speed (m/s). Daily PM_2.5_ concentration data were obtained from seven fixed-site monitoring stations located across the main urban area of Shijiazhuang, covering five administrative districts (Xinhua, Chang’an, Qiaoxi, Yuhua, and Gaoxin). These stations are distributed in areas with diverse land-use types, including residential, commercial, educational, and park zones. The spatial coverage of the monitoring network is considered representative of the population-weighted exposure to PM_2.5_ in the city’s built-up area. City-wide average concentrations were calculated to represent population-level ambient exposure. The spatial distribution of the air quality monitoring stations used in this study is shown in Supplementary Figure S1.

### 2.2 Calculation of AT

AT was calculated according to the Steadman AT formulation (18), which incorporates three meteorological variables: mean temperature, relative humidity, and wind speed. The formula is expressed as follows:


$$\begin{array}{l} \mathrm{AT}=-2.7+1.047T+0.2*(\frac{\mathrm{rh}}{100})*6.1094\\ {}*\exp(\frac{\left(17.625T\right)}{\left(243.04+T\right)})-0.65w \end{array}$$ (1)

Where AT denotes apparent temperature (°C), T represents daily mean temperature (°C), w is wind speed (m/s), and RH is relative humidity (%).

### 2.3 Definition of joint events

Low AT events were defined using a percentile-based approach based on the distribution of daily AT from 2014 to 2023 (19). AT below the 10th percentile were classified as mild low AT (T1: −5.32 °C < AT ≤ −3.43 °C), those below the 5th percentile as moderate low AT (T2: −6.95 °C < AT ≤ −5.32 °C), and those below the 2.5th percentile as extreme low AT (T3: AT ≤ − 6.95 °C).

PM_2.5_ pollution events were categorized into four levels: P1 (75 μg/m^3^ ≤ PM_2.5_ < 115 μg/m^3^), corresponding to the 24-h average limit specified in the Chinese Ambient Air Quality Standards (GB 3095–2012); P2 (115 μg/m^3^ ≤ PM_2.5_ < 150 μg/m^3^), corresponding to moderate pollution levels defined by the Chinese Air Quality Index (AQI) (20); P3 (150 μg/m^3^ ≤ PM_2.5_ < 203 μg/m^3^), corresponding to the AQI threshold for heavy pollution (21); and P4 (PM_2.5_ ≥ 203 μg/m^3^), defined as the 95th percentile based on local data from 2014 to 2023, representing extreme pollution levels.

When PM_2.5_ pollution and low AT events occurred simultaneously, the day was classified as a joint exposure event. Joint events were defined according to pollution level (P1–P4) and low AT intensity (T1–T3), yielding combinations such as P1T1, P1T2, P1T3, P2T1, P2T2, P2T3, P3T1, P3T2, P3T3, P4T1, P4T2 and P4T3(For example, P2T1 represents a joint event of moderate PM_2.5_ pollution and mild low AT).

### 2.4 Statistical analysis

#### 2.4.1 Association between apparent low temperature and circulatory emergency ambulance calls

DLNM was applied to evaluate the non-linear and lagged associations between AT and circulatory emergency ambulance calls (EACs). Considering that the number of emergency ambulance calls exhibited overdispersion (variance greater than the mean), a quasi-Poisson regression model was applied. The model was specified as follows (22–24):


$$\begin{array}{l} \text{logE}(Y_{t})=\alpha +\beta \cdot \text{basis}.\mathrm{AT}+\mathrm{ns}(\text{time},7\times 10)+\mathrm{ns}(\text{rhum},3)\\ +\gamma {\text{Holiday}}_{t}+\mathrm{\delta DO}W_{t}+\mathrm{ns}(\text{pollutants},3) \end{array}$$ (2)

where Yt is the EACs; *α* is the intercept; *β* and *γ* are regression model coefficients;basis AT represents the cross-basis function of AT and lag days; ns denotes a natural cubic spline; RH and pollutants (PM_2.5_ and O_3_) were included using natural cubic splines with 3 degrees of freedom (*df*); time was used to control for long-term trends; Holiday_t_ indicates public holidays; and DOW*t* represents the day of the week. The optimal model specification was determined using the Akaike Information Criterion (AIC) (25, 26). Lag effects of low AT were assessed over a lag period of 0–21 days.

In addition, attributable fraction (AF) analysis was conducted to further quantify the potential burden of non-optimal AT. The minimum-risk AT was used as the reference, and the total AF was calculated to estimate the cumulative impact of non-optimal AT on EACs. The AF was calculated as:


$$\mathrm{AFs}=\sum_{i=l0}^{L}\frac{\left(RR_{i}-1\right)}{(RR_{i}-1)+1}=1-\exp(-\sum_{i=l0}^{L}\beta x_{i})\;\;$$ (3)

where RRi is the relative risk at each exposure level compared to the baseline level, l_0_ and L represent the minimum and maximum lag days, respectively, *β*xi is the effect parameter at exposure level i.

All statistical analyses were performed using R software (version 4.3.2), with the mgcv and dlnm packages.

#### 2.4.2 Joint effects of low AT and PM_2.5_ on EACs

Given that the daily number of EACs (Yt) is count data and exhibited overdispersion (variance greater than the mean), a quasi-Poisson regression model was applied. The model was specified as:


$$\begin{array}{l} \log[E(\mathrm{Yt})]=\beta 0+\beta 1*\mathrm{cb}\_\mathrm{pm}+\beta 2*\mathrm{cb}\_\text{cold}+\beta 3\\ {}*\mathrm{cb}\_\text{joint}+\mathrm{ns}(\text{rhum},\mathrm{df}=3)+\mathrm{ns}(\text{date},\mathrm{df}=7*10)\\ +\text{weekdays}+\text{holiday} \end{array}$$ (4)

where Yt is the EACs; cb_pm , cb_cold and cb_joint represent the cross-basis matrices for PM_2.5_ events, low AT events, and joint events, respectively; ns(RH, *df* = 3) is the natural spline smoothing term for relative humidity; ns(date, *df* = 7 × 10) controls for long-term trends and seasonality (7 *df* per year, 70 df over the entire study period); weekdays and holiday represent day-of-week and public holiday indicators. The maximum lag period was set to 14 days. The selection of maximum lag days and degrees of freedom for spline functions was based on minimizing the AIC (25, 26).

To evaluate the interaction between low AT and PM_2.5_ pollution on EACs risk, the relative excess risk due to interaction (RERI) was calculated. The reference exposure for RERI calculation was defined as days with PM_2.5_ < 75 μg/m^3^ and AT > 10th percentile. A RERI > 0 indicates synergistic interaction, whereas a RERI < 0 indicates antagonistic interaction. The RERI was calculated as:


RERI=RR_joint−RR_pm_only−RR_cold_only+1 (5)

#### 2.4.3 Selection of maximum lag days

Based on considerations of model stability and exposure frequency, different maximum lag periods were applied for single-exposure and joint-exposure analyses. A longer lag period (0–21 days) was used for single apparent temperature (AT) analysis to fully capture the delayed effects of cold exposure. In contrast, a shorter lag period (0–14 days) was adopted for joint exposure analysis because some joint event categories (e.g., P4T3) occurred infrequently, and extending the lag period led to unstable effect estimates and substantial fluctuations in the lag-response curves.

The selection of maximum lag days was guided by the Akaike Information Criterion (AIC) minimization principle (25, 26). For the single AT model, the lowest AIC value was achieved at lag 21 days (23314.36); therefore, 0–21 days was used as the primary analysis window for the single-exposure analysis. For the joint exposure model (using P1T3 as an example), the AIC values for lags 14, 21, and 28 days were 22946.01, 22948.13, and 22949.41, respectively, with the minimum at lag 14 days (Supplementary Table S4). Accordingly, a 14-day maximum lag was selected as the primary analysis window for joint exposure analyses to ensure model stability and interpretability of the results.

Therefore, the use of different maximum lag periods reflected differences in exposure frequency and model stability rather than selective optimization of statistical significance.

To further evaluate the stability of the lag structure, extended lag-response analyses (up to lag 21 days) were performed for total circulatory emergency ambulance dispatches (Supplementary Figure S4).

#### 2.4.4 Sensitivity analyses

To assess the robustness of the findings, several sensitivity analyses were conducted for the single-factor apparent temperature (AT) effects, including alternative lag structures (0–14 and 0–28 days), different percentile thresholds for defining low AT exposure, and varying degrees of freedom for long-term trends and smoothing functions.

In the joint exposure analysis, we selected the combined event combinations based on the AIC values from Supplementary Table S5. According to the minimum AIC, P1T3 and P2T2 were chosen as representative joint event combinations. The FDR approach was selected to balance the risks of type I and type II errors given the relatively large number of subgroup comparisons. To account for multiple comparisons in subgroup analyses, *p* values were adjusted using the Benjamini–Hochberg method to control the false discovery rate (FDR).

Representative exposure scenarios were selected for sensitivity analyses, including extreme low temperature (T3) and typical joint exposure combinations (P1T3 and P2T2), to examine the stability of the main findings under different model specifications.

In addition, to evaluate potential seasonal effect modification, we conducted a season-stratified sensitivity analysis restricted to winter (December to February), when low apparent temperature and elevated PM_2.5_ levels were more prevalent. Using the same model structure as the main analysis, the joint effects and additive interactions (RERI) between low AT and PM_2.5_ were re-estimated.

#### 2.4.5 Other statistical analyses

Additional statistical analyses were performed using R software (version 4.3.2). The Shapiro–Wilk test indicated that air pollutant concentrations, meteorological variables, and EACs data were non-normally distributed; therefore, these variables were summarized using the median and interquartile range [M (*P*_25_, *P*_75_)].

## 3 Results

### 3.1 Basic results

A total of 82,714 EACs records for circulatory emergency ambulance calls were collected in this study. Regarding sex distribution, males accounted for approximately 55.7% of all EACs, while females accounted for 44.3%. In terms of age structure, the proportion of patients aged 65 years and older was higher than that of patients younger than 65 years.

Marked differences were observed in the distributional characteristics of daily mean temperature and AT. The median daily mean temperature was 16.0 °C, with a relatively concentrated distribution and limited variability, AT exhibited a substantially wider range of daily fluctuations (Table 1).

**Table 1:** Descriptive statistics of daily EACs, meteorological variables, and air pollutants in Shijiazhuang City, China from 2014 to 2023.

Variable	Mean	Min	*P*_25_	Median	*P*_75_	Max
Circulatory system diseases	22.65	4	18	22	26	68
Cerebrovascular disease	8.36	0	6	8	10	26
Heart diseases	5.06	0	3	5	7	21
Hypertension	3.71	0	2	3	5	22
Male	12.6	1	10	12	15	38
Female	10.02	1	7	10	12	35
<65 years	8.79	0	6	9	11	34
≥65 years	13.86	2	11	13	17	43
Average daily temperature/°C	14.95	−10.8	5	16	24.7	35.8
AT/°C	13.98	−15.4	2.27	14.71	25.59	36.39
Average relative humidity/%	55.84	7	40	56	72	100
Wind speed (m/s)	1.89	0.3	1.3	1.8	2.3	6.5
PM_2.5_	73.24	6.3	31	51.4	89.62	625.3
O_3_	93.90	1.9	49.25	82.8	128.18	1156.25

### 3.2 Effects of AT on EACs for circulatory system diseases

#### 3.2.1 AF analysis of low AT

AF analysis indicated that, in the circulatory system diseases, 4.55% (95% CI, 2.39%–6.10%) of EACs for circulatory system diseases were attributable to low AT. Across cold intensity categories, T1 contributed the largest attributable burden (AF = 2.14, 95% CI: 1.02%–2.94%), exceeding that of T3 (AF = 1.25, 95% CI: 0.74%–1.62%) and T2 (AF = 1.16, 95% CI: 0.63%–1.54%).

In population-specific analyses, the overall attributable burden was comparable between males (AF = 4.54%) and females (AF = 4.90%); however, individuals aged ≥65 years (AF = 4.82%) experienced a higher attributable burden than those aged <65 years (AF = 4.11%). In disease-specific analyses, heart disease exhibited the highest attributable fraction (AF = 5.22, 95% CI: 2.00%–7.14%), followed by cerebrovascular diseases (AF = 4.82, 95% CI: 2.22%–6.56%) and hypertension (AF = 3.70, 95% CI: −3.06%–6.97%) (Table 2).

**Table 2:** Attributable risk analysis of apparent low temperature for emergency call volume due to circulatory system diseases.

Outcome	Apparent low temperature	T1	T3	T2
Circulatory system diseases	4.55% (2.39–6.10%)	2.14% (1.02–2.94%)	1.25% (0.74–1.62%)	1.16% (0.63–1.54%)
Cerebrovascular disease	5.22% (2.00–7.14%)	2.46% (0.98–3.47%)	1.42% (0.67–1.87%)	1.33% (0.55–1.80%)
Ischemic heart diseases	3.70%(−3.06-6.97%)	1.66% (−1.86–3.38%)	1.08% (−0.51–1.83%)	0.96% (−0.68–1.76%)
Hypertension	8.04%(5.78%- ~ 9.10%)	3.95% (2.75–4.51%)	2.08% (1.56–2.32%)	2.02% (1.47–2.27%)
Male	4.54% (1.68–6.41%)	2.09% (0.60–3.08%)	1.28% (0.61–1.71%)	1.17% (0.47–1.62%)
Female	4.90% (1.79–6.83%)	2.35% (0.77–3.35%)	1.31% (0.55–1.77%)	1.24% (0.47–1.71%)
<65 years	4.11% (0.27–6.44%)	1.94% (−0.02–3.14%)	1.12% (0.19–1.68%)	1.05% (0.10–1.62%)
≥65 years	4.82% (2.22–6.56%)	2.26% (0.91–3.17%)	1.33% (0.72–1.73%)	1.23% (0.60–1.65%)

### 3.3 The impact of joint events on circulatory emergency ambulance calls (EACs)

#### 3.3.1 Descriptive characteristics of joint events

A total of 183 joint exposure days were identified during the study period. The proportion of joint events relative to all study days ranged from 0.96% for P1T1 events to 0.11% for P4T3 events.

Because only four P4T3 exposure days were identified during the study period, the corresponding estimates were considered exploratory and interpreted cautiously because of potential instability and wide confidence intervals (Table 3).

**Table 3:** Basic information on joint low AT and PM_2.5_ events.

Definition	Event days (*N*)	The percentage of total events (%)
P4T1	35	19.12
P1T1	33	18.03
P2T1	21	11.48
P1T2	19	10.38
P3T1	17	9.29
P1T3	15	8.20
P2T2	10	5.46
P3T2	10	5.46
P3T3	7	3.83
P2T3	6	3.28
P4T2	6	3.28
P4T3	4	2.19
Total	183	100.00

#### 3.3.2 Single-day lag effects of joint exposure events on circulatory emergency ambulance calls (EACs)

Joint exposure events exhibited significant associations with multiple health outcomes across different lag days, with pronounced lag structures and substantial population heterogeneity.

In the circulatory system diseases, peak risks associated with joint exposure events generally occurred between lags 3 and 12 days. For example, the highest risk for P1T3 events was observed at lag 3 (*RR* = 1.155, 95% CI: 1.028–1.299), whereas P1T2 events showed the strongest association at lag 11 (*RR* = 1.162, 95% CI: 1.052–1.284).

Sex-stratified analyses indicated greater susceptibility among males. Under P1T2 exposure, males experienced the highest risk at lag 11 (*RR* = 1.259, 95% CI: 1.114–1.423), whereas females exhibited the strongest association under P1T1 exposure at lag 12 (*RR* = 1.131, 95% CI: 1.009–1.267). Age-stratified analyses revealed that individuals aged ≥65 years showed significant risks at lags 4, 7, 10, and 11 under P1T3 exposure, while among those aged <65 years, the highest risk was observed under P4T2 exposure at lag 8 (*RR* = 1.426, 95% CI: 1.111–1.831).

**Figure 1:**
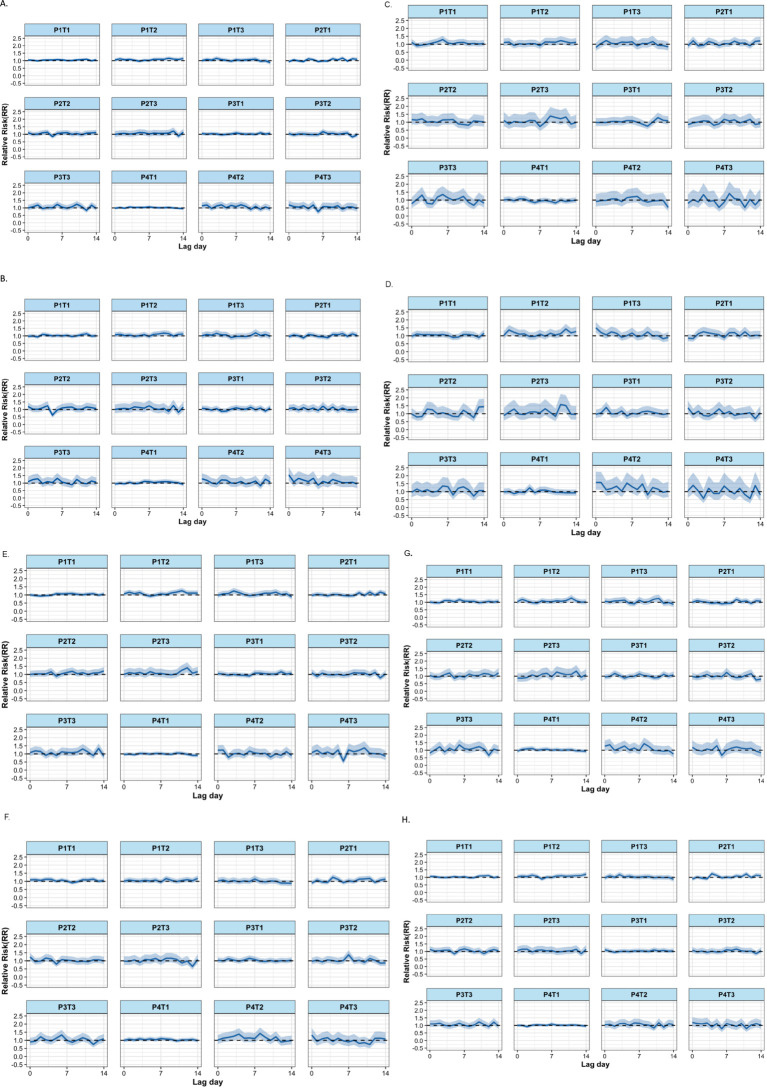
Single-day lag effects of joint exposure events on EACs. **(A)** Circulatory system diseases; **(B)** cerebrovascular diseases; **(C)** heart diseases; **(D)** hypertension; **(E)** males; **(F)** females; **(G)** < 65 years; **(H)** ≥ 65 years.

Disease-specific analyses further demonstrated heterogeneity in susceptibility. Patients with hypertension exhibited the highest risk under P4T2 exposure (*RR* = 1.583, 95% CI: 1.114–2.249). The highest relative risk among patients with heart disease was observed under P1T1 exposure (*RR* = 1.298, 95% CI: 1.101–1.529), whereas patients with cerebrovascular diseases showed the strongest association under P3T3 exposure (*RR* = 1.307, 95% CI: 1.027–1.664) (Figure 1).

To further evaluate the stability of the lag structure, extended lag-response analyses up to lag 21 days were additionally conducted for different disease subtypes and population subgroups under joint exposure conditions (Supplementary Figure S4). Compared with the primary analyses using lag 0–14 days (Figure 1), the overall temporal patterns of the joint exposure effects remained generally consistent during the early lag period, with elevated risks mainly observed within the first 3–7 days after exposure. However, beyond lag 14 days, several joint exposure categories exhibited progressively wider confidence intervals and greater fluctuations in effect estimates, particularly for rare joint exposure categories with sparse observations such as P4T3. These findings suggest that extending the lag period beyond 14 days may reduce model stability and interpretability, thereby supporting the use of lag 0–14 days as the primary analysis window for joint exposure analyses.

#### 3.3.3 Cumulative lag effects of joint exposure events on circulatory emergency ambulance calls (EACs)

For most joint exposure events, the risk of circulatory emergency ambulance calls (EACs) increased significantly. The cumulative lag analysis indicated that, in the overall population, the P1T3 joint event was associated with the highest relative risk (*RR* = 1.146, 95% CI: 1.088–1.207).

Marked heterogeneity in risk patterns was observed across population subgroups and disease categories. In sex-stratified analyses, males exhibited the highest risk under the P2T2 joint event (*RR* = 1.107, 95% CI: 1.047–1.173), whereas females showed the strongest association under the P1T2 joint event (*RR* = 1.155, 95% CI: 1.074–1.242). Age-stratified analyses demonstrated that both individuals aged <65 years and those aged ≥65 years reached peak risks during P1T3 joint event, with RRs of 1.159 (95% CI: 1.071–1.254) and 1.136 (95% CI: 1.068–1.210).

Disease-specific analyses further revealed that patients with hypertension experienced the strongest association during P1T2 joint events (*RR* = 1.249, 95% CI: 1.112–1.403). Similarly, heart disease showed the highest cumulative risk under P1T2 joint events (*RR* = 1.179, 95% CI: 1.055–1.317), whereas cerebrovascular diseases were most strongly associated with P1T3 joint events (*RR* = 1.157, 95% CI: 1.068–1.252).

**Figure 2:**
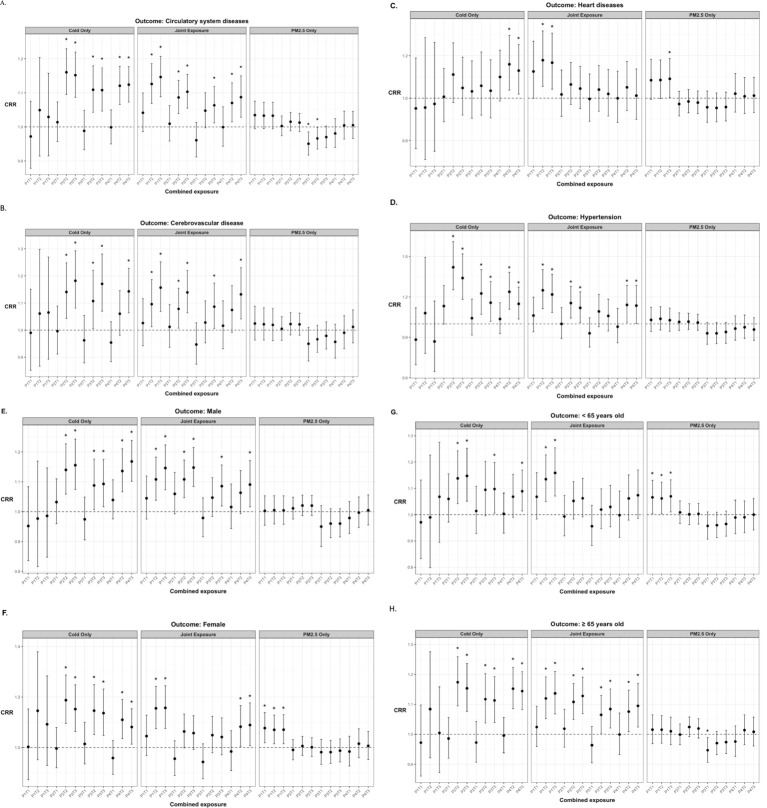
Cumulative relative risks of joint exposure events over lag days 0–14. *p < 0.05. **(A)** Circulatory system diseases; **(B)** cerebrovascular diseases; **(C)** heart diseases; **(D)** hypertension; **(E)** males; **(F)** females; **(G)** <65 years; **(H)** ≥65 years.

Notably, when PM_2.5_ concentrations increased to the P3 level, the joint effects were attenuated, with the relative risks for some outcomes approaching or falling below unity. This pattern suggests that under higher PM_2.5_ exposure levels, the additional effect of low AT may be partially masked (Figure 2).

#### 3.3.4 Interaction effects

The interaction analysis revealed pronounced disease-specific patterns and population heterogeneity in the joint effects of low AT and PM_2.5_ on the risk of circulatory emergency ambulance calls (EACs). In the overall population, most P2-series joint events exhibited negative interactions, with P2T2 showing statistical significance (RERI = −0.089, 95% CI: −0.153 ~ −0.024), indicating that the combined effect was smaller than the sum of the independent effects of the two exposures. Although P1-series joint events tended to show positive interactions, none reached statistical significance.

Sex-stratified analyses demonstrated that males exhibited significant positive interactions under P1T3 (RERI = 0.156, 95% CI: 0.007 ~ 0.305) and P3T1 (RERI = 0.080, 95% CI: 0.014 ~ 0.146), suggesting synergistic effects. In contrast, females showed significant negative interactions under P2T2 (RERI = −0.130, 95% CI: −0.221 ~ −0.039) and P2T3 (RERI = −0.096, 95% CI: −0.189 ~ −0.003). Age-stratified results indicated that individuals aged ≥65 years exhibited a significant negative interaction under the P2T2 joint event (RERI = −0.090, 95% CI: −0.168 ~ −0.012), whereas in those aged <65 years, P2-series joint events consistently showed negative interaction trends that did not reach statistical significance.

**Figure 3:**
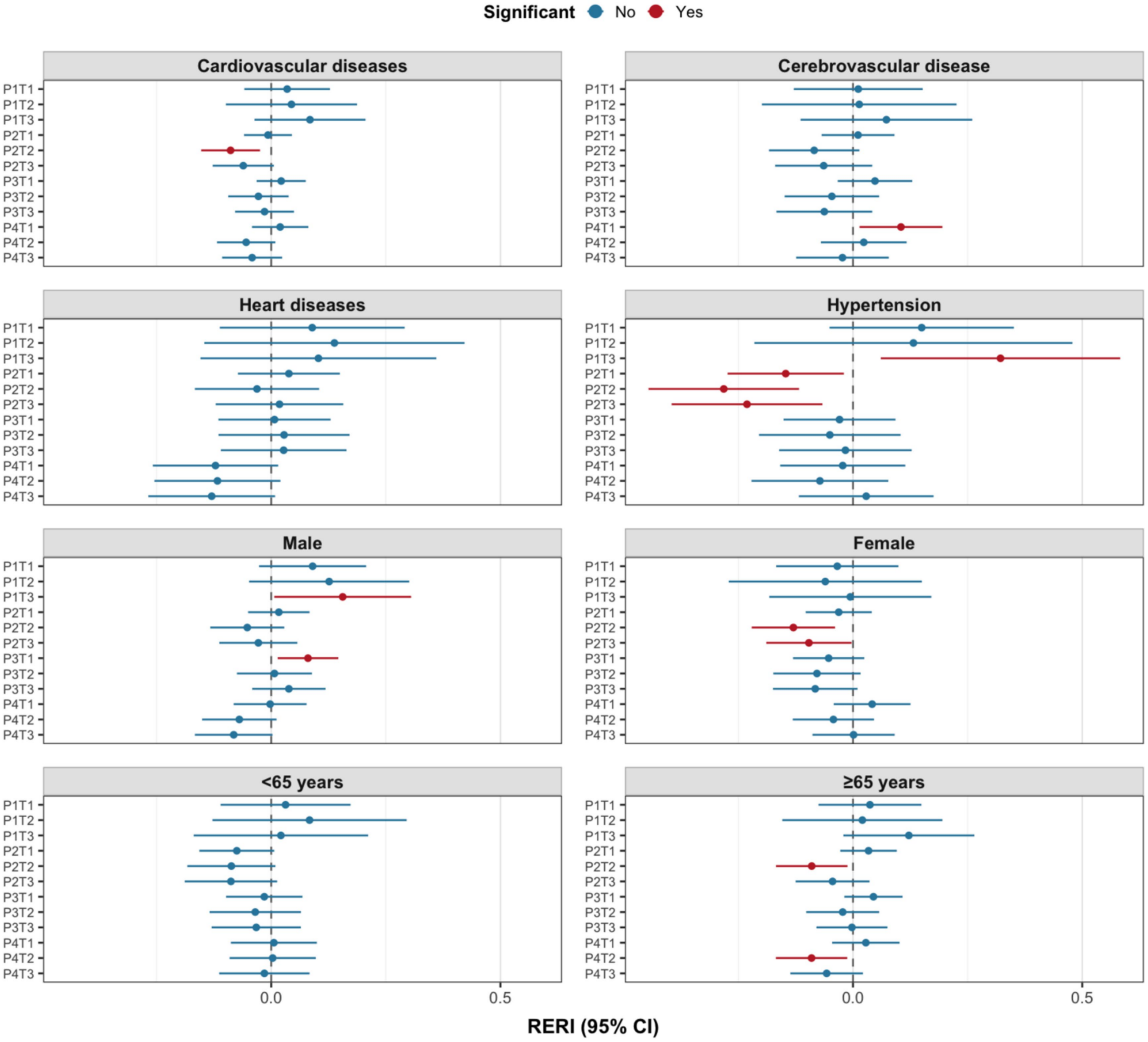
Relative excess risk due to interaction (RERI) for joint exposure events of low apparent temperature and PM_2.5_ on emergency ambulance calls. Error bars indicate 95% confidence intervals. RERI is a unitless measure representing the additional risk beyond the sum of individual effects.

Disease-specific analyses further showed that patients with hypertension experienced a significant positive interaction under the P1T3 joint event (RERI = 0.322, 95% CI: 0.061 ~ 0.583), indicating a synergistic effect between low AT and moderate PM_2.5_ pollution. Among patients with heart diseases, P4-series joint events generally exhibited negative interaction trends, although not all reached statistical significance. For cerebrovascular diseases, interactions varied in direction: a significant positive interaction was observed under the P4T1 joint event (RERI = 0.105, 95% CI: 0.014 ~ 0.195), whereas a negative interaction trend was noted under the P2T2 joint event (RERI = −0.085, 95% CI: −0.183 ~ 0.014), although this did not reach statistical significance but showed a clear tendency (Figure 3).

#### 3.3.5 Sensitivity analyses

The association between apparent temperature (AT) and emergency ambulance calls (EACs) remained robust across multiple sensitivity analyses. For single AT effects, alternative lag structures, exposure definitions, and model parameter settings resulted in only minor changes in the estimated effects (Supplementary Table S2).

Similarly, the findings for joint exposure analyses were generally consistent under different lag specifications, model settings, and additional covariate adjustments (Table 4).

**Table 4:** Sensitivity analyses for joint effects of low AT and PM_2.5_.

Analysis type	Specification	CRR (P1T3)	CRR (P2T2)
Main model	Lag 0–14	1.146 (1.088–1.207)	1.087 (1.039–1.136)
Lag	0–21	1.089 (1.027–1.156)	1.082 (1.031–1.136)
	0–28	1.145 (1.087–1.206)	1.085 (1.037–1.135)
Adjustment	+ O3	1.148 (1.090–1.209)	1.087 (1.039–1.137)
Model *df*	Time *df* = 6	1.132 (1.077–1.190)	1.088 (1.042–1.136)
	Time *df* = 8	1.138 (1.079–1.201)	1.078 (1.029–1.129)
	RH *df* = 4	1.145 (1.087–1.206)	1.085 (1.037–1.135)
	RH *df* = 5	1.145 (1.087–1.206)	1.085 (1.037–1.135)

After FDR correction, the extreme joint exposure (P1T3) remained statistically significant in most subgroups (FDR *p* < 0.05); whereas the moderate exposure (P2T2) showed weaker effects and its adjusted *p*-values did not reach significance in females, individuals aged <65 years, and the heart disease subgroup (FDR *p* = 0.122, 0.246, and 0.297, respectively). Overall, the main conclusions were not altered by multiple comparisons (Table 5).

**Table 5:** Subgroup analyses with FDR-adjusted *p*-values.

Subgroup	P1T3 CRR (95% CI)	*p*	FDR_p_	P2T2 CRR (95% CI)	*p*	FDR_p_
Male	1.146 (1.073–1.223)	<0.001	<0.001	1.108 (1.047–1.173)	<0.001	0.002
Female	1.157 (1.076–1.245)	<0.001	0.001	1.064 (0.999–1.133)	0.054	0.122
<65 years	1.159 (1.071–1.254)	<0.001	0.002	1.052 (0.983–1.126)	0.142	0.246
≥65 years	1.136 (1.068–1.210)	<0.001	<0.001	1.108 (1.050–1.170)	<0.001	0.002
Cerebrovascular disease	1.157 (1.068–1.252)	<0.001	0.002	1.078 (1.007–1.154)	0.03	0.08
Heart diseases	1.167 (1.043–1.306)	0.007	0.028	1.064 (0.970–1.168)	0.187	0.297
Hypertension	1.218 (1.085–1.368)	<0.001	0.005	1.154 (1.043–1.278)	0.006	0.023

Additional winter-only analyses suggested that the joint effects of low AT and PM_2.5_ were generally stronger during winter than in the full-year analysis for several outcomes. In particular, stronger additive interactions were observed for ischemic heart disease and hypertension under selected joint exposure scenarios. For some moderate joint exposure categories, the relative excess risk due to interaction (RERI) shifted from antagonistic in the annual model to synergistic in winter (Supplementary Table S3).

## 4 Discussion

This study, based on emergency dispatch, meteorological, and air pollution data from Shijiazhuang during 2014–2023, systematically evaluated the effects of apparent low temperature and its combined exposure with PM_2.5_ on the risk of circulatory emergency ambulance calls. Our results indicate that apparent low temperature is significantly associated with increased risk of circulatory emergency ambulance calls, exhibiting a lagged effect. Furthermore, when apparent low temperature and PM_2.5_ co-occurred, the risk of circulatory emergency ambulance calls increased further, with the highest risk observed under the P1T3 joint event definition (*RR* = 1.146, 95% CI: 1.088–1.207). This study is among the few that utilize high temporal resolution emergency data to systematically assess the acute circulatory health impacts of combined low-temperature and PM_2.5_ exposure, providing new epidemiological evidence on the effects of compound environmental exposures on circulatory health.

In the analysis of individual environmental factors, we found a significant association between apparent low temperature and increased risk of circulatory emergency ambulance calls, with a lagged effect over several days. These findings are consistent with previous temperature–health studies (27, 28), which reported that cold exposure has a significant and persistent delayed effect on circulatory mortality, suggesting that low temperatures may adversely affect the circulatory system through cumulative physiological stress.

The attributable fraction analysis further revealed that approximately 4.55% (95% CI: 2.39%–6.10%) of circulatory emergency ambulance calls were attributable to low apparent temperature, with mild cold (T1) contributing the largest burden (AF = 2.14, 95% CI: 1.02%–2.94%). This finding highlights that even non-extreme cold conditions may have a substantial public health impact, particularly among older adults (AF = 4.82%) and patients with heart disease (AF = 5.22%).

Compared with single-factor exposure, this study focuses on the joint effects of apparent low temperature and PM_2.5_ pollution. Our results show that, across most joint event definitions, risk of circulatory EACs was markedly elevated, indicating that the co-occurrence of cold and air pollution may substantially increase the short-term risk of circulatory emergency ambulance calls. Similar findings have been reported in other regions. For example, a study conducted in Shanghai found that joint exposure to PM_2.5_ and cold waves was significantly associated with cause-specific mortality (29). Another study in rural southern Xinjiang, China, reported that combined exposure to low temperature and high pollutant concentrations increased the risk of coronary heart disease hospitalization by 1.53 times (27). Additionally, research in high-altitude areas showed that the interaction of cold waves and high PM_2.5_ concentrations could account for 14%–49% of ischemic heart disease hospitalizations attributable to joint exposure (30). A recent study in cold regions also reported that simultaneous exposure to extreme low temperatures and PM_2.5_ significantly increased the risk of acute myocardial infarction (28). Collectively, these findings are generally consistent with our results, further supporting the potential synergistic effect of adverse meteorological conditions and air pollution in increasing circulatory event risk.

Our findings are generally consistent with previous studies conducted under different climatic and environmental settings. Several studies have reported that ambient temperature may substantially modify the health effects of air pollution, particularly during periods of thermal stress. For example, a recent study from Istanbul found that the associations between ambient air pollutants and COPD hospitalizations became stronger under both low- and high-temperature conditions, highlighting the important modifying role of temperature on pollution-related health risks (31). Similarly, studies from other regions of China have reported significant interactive effects between PM_2.5_ and extreme temperatures on mortality and circulatory outcomes, suggesting that compound environmental exposures may exert complex nonlinear health effects (32, 33). In addition, previous circulatory studies have shown that low temperature may enhance PM_2.5_-related circulatory risks through increased oxidative stress, systemic inflammation, endothelial dysfunction, and sympathetic nervous system activation (34). These findings are broadly consistent with the stronger joint effects observed during the cold season in the present study. However, regional differences in climatic background, pollution sources, PM_2.5_ chemical composition, population susceptibility, and behavioral adaptation may contribute to heterogeneity in observed exposure–response relationships across studies.

From an environmental process perspective, wintertime stable atmospheric layers, weak wind conditions, and low temperatures often favor pollutant accumulation near the surface, creating a “low temperature plus high pollution” scenario (35). Under such conditions, populations may be simultaneously exposed to adverse meteorological factors and elevated pollution levels, thereby increasing the risk of circulatory emergency ambulance calls (30, 36, 37). In our study, similar trends were observed in the hypertension subgroup, for instance, under the P1T2 joint event definition, emergency risk further increased (*RR* = 1.217, 95% CI: 1.089–1.360), suggesting that individuals with underlying circulatory conditions may be more susceptible to compound environmental exposures.

In addition, this study observed heterogeneous interaction patterns between apparent low temperature and PM_2.5_ across different subgroups, characterized by enhanced synergistic effects in some populations and antagonistic effects in others. This heterogeneity may be jointly driven by physiological differences and behavioral factors. In terms of sex, males exhibited stronger synergistic effects under certain combined exposure scenarios, which may be related to their higher levels of outdoor activity, greater occupational exposure, and a higher burden of circulatory risk factors (38, 39). In contrast, the antagonistic effects observed among females may be associated with stronger risk-avoidance behaviors as well as the potential protective role of estrogen on vascular function (40, 41). Within subgroups defined by underlying diseases, patients with hypertension showed more pronounced synergistic effects, possibly due to impaired vascular regulation and increased blood pressure variability (42), rendering them more susceptible to temperature fluctuations and air pollution. By comparison, the antagonistic effects observed among patients with heart disease may be attributable to their heightened health risk awareness and more frequent medical interventions (43), such as reduced outdoor activity and optimized medication management under adverse environmental conditions, which may partially mitigate the combined impact of environmental exposures. These differences may also reflect the complex nonlinear interactions between environmental exposures and individual susceptibility, whereby different populations may exhibit varying directions and magnitudes of health effects under different exposure levels. The observed antagonistic interactions may also reflect statistical factors or exposure measurement limitations, in addition to potential biological mechanisms, and therefore should be interpreted with caution. The extended lag analyses further demonstrated that the main joint exposure patterns observed within lag 0–14 days remained generally stable, whereas longer lag specifications introduced greater uncertainty and instability in the estimates, especially for infrequent joint exposure categories.

From a biological mechanism perspective, low temperature and PM_2.5_ may jointly affect the circulatory system through distinct but interconnected pathways. PM_2.5_ particles can enter the respiratory tract and cross alveolar membranes into the bloodstream, triggering systemic inflammation, oxidative stress, and endothelial dysfunction, thereby promoting atherosclerosis (34). Concurrently, cold exposure can activate the sympathetic nervous system, inducing peripheral vasoconstriction, elevated blood pressure, and increased blood viscosity, which may enhance thrombosis risk (38, 44). Notably, patients with hypertension often exhibit impaired vascular regulatory function and increased arterial stiffness (45), making them more sensitive to environmental changes. Mild cold exposure may induce elevations in blood pressure (46) without being sufficient to trigger protective behavioral responses, while low-level PM_2.5_ exposure can further provoke inflammatory responses and endothelial dysfunction (47). The joint effects of these exposures may therefore act synergistically, potentially triggering acute circulatory events even under relatively moderate environmental conditions. This may help explain why the highest risk was observed under the P1T2 scenario (mild pollution combined with moderate cold). Compared with extreme conditions, moderate environmental stressors may be insufficient to prompt protective behaviors (staying indoors or using heating), resulting in sustained exposure (34, 44, 48). However, these conditions can still induce measurable physiological responses, including increased blood pressure and systemic inflammation. Together, these processes may elevate the risk of acute circulatory events, particularly among susceptible populations such as patients with hypertension. In addition, this pattern is consistent with a nonlinear exposure–response relationship, in which moderate environmental stressors may exert disproportionately greater health effects in susceptible individuals than more extreme conditions, possibly due to differences in exposure duration and behavioral adaptation. Nevertheless, caution is warranted when interpreting the effects of extremely rare compound exposure scenarios. Although the P4T3 category (extreme PM_2.5_ pollution combined with extreme apparent cold) may represent a particularly important compound exposure scenario from a climate change and public health perspective, only four such exposure days were identified during the study period. Consequently, the corresponding estimates were relatively unstable and accompanied by wide confidence intervals. Therefore, these findings should be interpreted cautiously.

Notably, under certain joint event definitions, our study observed a negative interaction between apparent low temperature and PM_2.5_, meaning that their combined effect was lower than the sum of their individual effects. This phenomenon has also been reported in environmental epidemiology studies. For example, some studies indicate that the health effects of temperature and air pollution do not always follow a simple additive pattern but may exhibit complex interaction modes (49). A study on pneumonia mortality also reported an antagonistic effect of joint cold wave and PM_2.5_ exposure, with a negative relative excess risk, suggesting that antagonistic interactions may occur under certain environmental conditions (50). Additionally, multi-region studies have reported that, under high pollution conditions, the marginal enhancing effect of temperature on health risks may be limited (30). Multiple mechanisms may contribute to this negative interaction. First, during extreme cold, people may reduce outdoor activity or adopt protective measures, thereby decreasing actual pollutant exposure. Second, in highly polluted environments, inflammatory and oxidative stress pathways may approach saturation, potentially limiting the additional effect of cold exposure on the circulatory system (51). Third, extreme cold may reduce metabolic rate or alter hemodynamic status, partially delaying the expression of pollutant toxicity (52). Therefore, the health effects of apparent low temperature and PM_2.5_ may follow complex, nonlinear interaction patterns (41), posing new challenges for public health interventions. In addition, limited statistical power for some joint exposure categories and non-differential exposure misclassification arising from fixed-site monitoring data may partly explain the observed antagonistic interactions. This non-linear pattern may reflect behavioral adaptation, depletion of susceptible individuals, or saturation of inflammatory pathways under extremely high pollution conditions.

Season-specific analyses further suggested that winter may amplify the adverse joint effects of low apparent temperature and PM_2.5_. This may be related to more frequent concurrent exposure, indoor/outdoor behavioral changes, and increased physiological vulnerability during cold seasons. These findings indicate that season should be considered when developing compound environmental risk warning systems. Seasonal variation in PM_2.5_ chemical composition may also partly explain the stronger joint effects observed during the cold season. In northern China, wintertime PM_2.5_ is characterized by higher proportions of secondary inorganic aerosols, particularly nitrate and sulfate components, largely due to coal combustion, stagnant meteorological conditions, and enhanced secondary formation processes (53, 54). These components have been associated with increased oxidative stress, systemic inflammation, and endothelial dysfunction, which may further amplify the circulatory effects of low-temperature exposure. However, because PM_2.5_ chemical composition data were unavailable in the present study, this hypothesis requires further investigation.

From a public health perspective, our findings have important implications. With the increasing frequency of extreme weather events and air pollution under climate change, the health impacts of compound environmental exposures are gaining attention. Our study suggests that during cold seasons, simultaneous exposure to low temperature and air pollution may significantly increase the risk of acute circulatory events. Establishing early warning systems for compound environmental health risks and implementing targeted interventions for vulnerable populations such as the older adults and individuals with hypertension may help mitigate the incidence of acute circulatory events during winter pollution episodes.

This study has several strengths. First, emergency dispatch data can capture short-term changes in acute health events, offering high sensitivity for assessing the short-term health effects of environmental exposures. Second, apparent temperature was used as the primary exposure indicator, integrating temperature, humidity, and wind speed, thereby more accurately reflecting actual human thermal perception. Additionally, multiple joint event definitions were constructed to systematically evaluate the health effects of compound environmental exposures.

However, some limitations remain. First, regarding exposure assessment, this study relied on city-wide average concentrations derived from fixed-site monitoring stations, which may not accurately reflect individual-level exposure. Because individual activity patterns were unavailable, some degree of spatial exposure misclassification is unavoidable, particularly given the substantial intra-urban heterogeneity of PM_2.5_ concentrations in Shijiazhuang. In addition, behavioral changes during periods of extreme cold or severe pollution, such as reduced outdoor activity, may further contribute to exposure measurement error. Second, as an ecological time-series study, we were unable to obtain information on individual behavioral patterns or underlying health conditions. In addition, other potential individual-level confounders—such as smoking status, body mass index (BMI), and medication use—were not accounted for; therefore, residual confounding cannot be completely ruled out. Third, the percentile-based definition of low temperature (T1–T3) in this study is location-specific and may not reflect universally applicable thresholds. Moreover, as the analysis was based on data from a single city, the generalizability of the findings may be limited and may not extend to regions with warmer climates, lower pollution levels, or higher altitudes, and multi-center studies are warranted to enhance generalizability. Fourth, this study did not consider the chemical composition of PM_2.5_, which may partly explain heterogeneity in the observed health effects, as different components may have varying levels of toxicity. Fifth, due to the small sample size for the combined extreme exposure category (P4T3), it was excluded from the analysis, which may introduce some uncertainty in estimating effects under extreme conditions. Therefore, the effect estimates and joint exposure classifications observed in this study should be interpreted with caution when compared across regions with different climatic conditions or population adaptive capacities.

In conclusion, this study demonstrates that combined exposure to apparent low temperature and PM_2.5_ is significantly associated with increased risk of circulatory emergency events, suggesting that compound environmental exposures may have important impacts on circulatory health. In the context of ongoing climate change and persistent air pollution, strengthening research on multi-environmental factor interactions and incorporating these findings into public health early warning and intervention systems are crucial for reducing the circulatory disease burden.

## Data Availability

The datasets used in this study are subject to institutional restrictions and are not publicly available. Access to the data requires permission from the relevant authorities. Requests to access these datasets should be directed to MG, gmyguan@126.com.
