# Supplementary material for: Combined effects of apparent low temperature and PM2.5 pollution on circulatory emergency ambulance calls: a time-series analysis in Shijiazhuang, China
Source: Front Public Health. 2026 Jul 7;14:1838363. doi: 10.3389/fpubh.2026.1838363 (PMC13385666; doi:10.3389/fpubh.2026.1838363)
Supplement: Supplementary file 1 [file Data_sheet_1.pdf]

# Combined effects of apparent low temperature and $PM_{2.5}$ pollution on cardiovascular emergency ambulance calls: A time-series analysis in Shijiazhuang, China

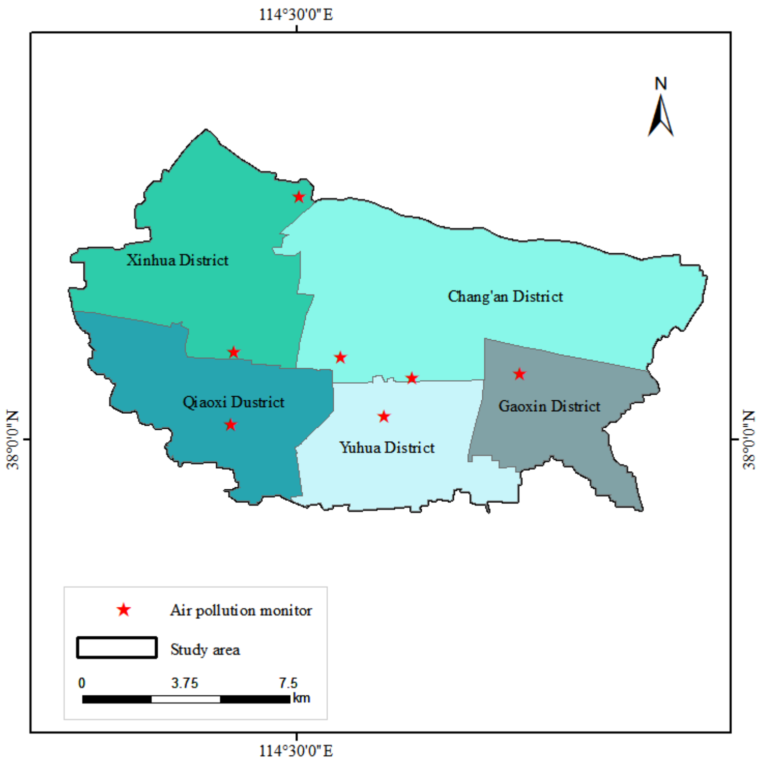


**Figure S1** Spatial distribution of air quality monitoring stations in Shijiazhuang.

The figure shows the locations of fixed-site air quality monitoring stations used in this study across the urban districts of Shijiazhuang.

## 2.2.Effects of AT on EACs for Circulatory system diseases

### 2.2.2. Effects of AT on EACs for Circulatory system diseases

The single-day lag analyses of AT on the risk of EACs for circulatory system diseases and its subgroups indicated a clear time-dependent pattern. Low AT exhibited lagged effects on EACs for overall circulatory system diseases, as well as among females, males, individuals aged <65 years and ≥65 years, and patients with cerebrovascular disease, hypertension, and heart disease, with effect estimates increasing as lag days progressed. In contrast, the effects of low AT on EACs for cerebrovascular disease and hypertension showed more acute patterns,

with the strongest effects occurring on the current day and gradually attenuating with increasing lag days (**Figure S2**).

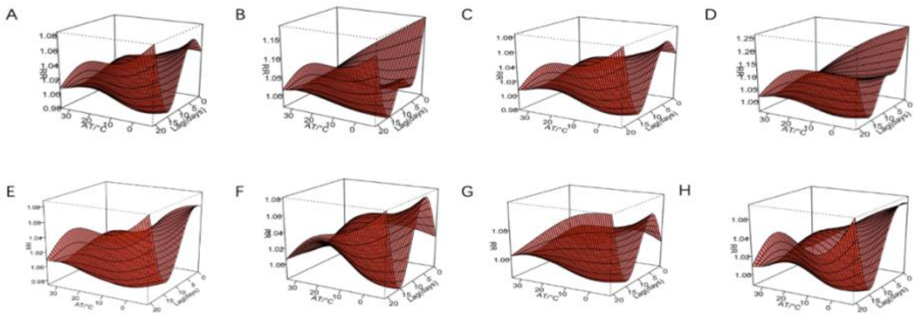


A：Circulatory system diseases；B: Cerebrovascular disease；C：Heart diseases；D：Hypertension；E：Male；F：Female；G：<65 years；H：≥65 years

**Figure S2** Single-day lag effects of apparent temperature on emergency call volume for circulatory diseases in Shijiazhuang City from 2014 to 2023

### 2.2.3. Single-Day Lag Effects of Low AT on EACs for Circulatory system diseases

Across the three low-temperature thresholds (T1–T3), significant lagged associations with emergency ambulance calls (EACs) for circulatory diseases were consistently observed. Overall, the effects generally appeared within several days after exposure and persisted for approximately one week. The peak effects tended to occur within 1–5 days, with relative risks (RRs) around 1.02–1.03.

Sex-stratified analyses revealed a consistent pattern: males exhibited earlier and stronger effects, typically on the same day or within the first few lag days (*RR*=1.04 at lag 0–1), whereas females showed delayed effects, with significant associations emerging mainly at lag 6–9 days and peaking around lag 8.

Age-stratified analyses indicated that individuals aged ≥65 years were more vulnerable, with significant effects occurring earlier (generally at lag 2–4 days; peak RR= 1.03). In contrast, the <65 years group showed weaker or delayed associations, and no significant effects were observed under T1.

Among the three thresholds, T3 showed a relatively longer lag structure (lag 2–9 days)

and a slightly higher peak estimate (*RR* = 1.026, 95% *CI*: 1.006–1.046), suggesting a more persistent impact under more extreme cold conditions **(Figure S3).**

A.

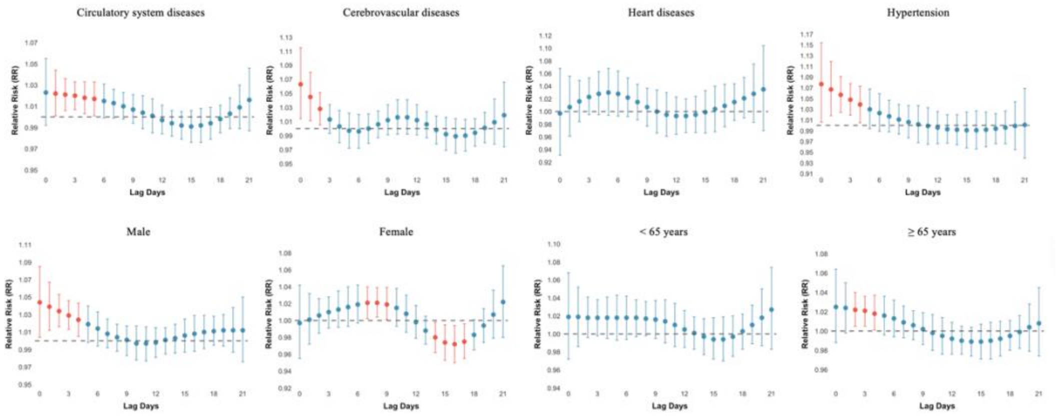


B.

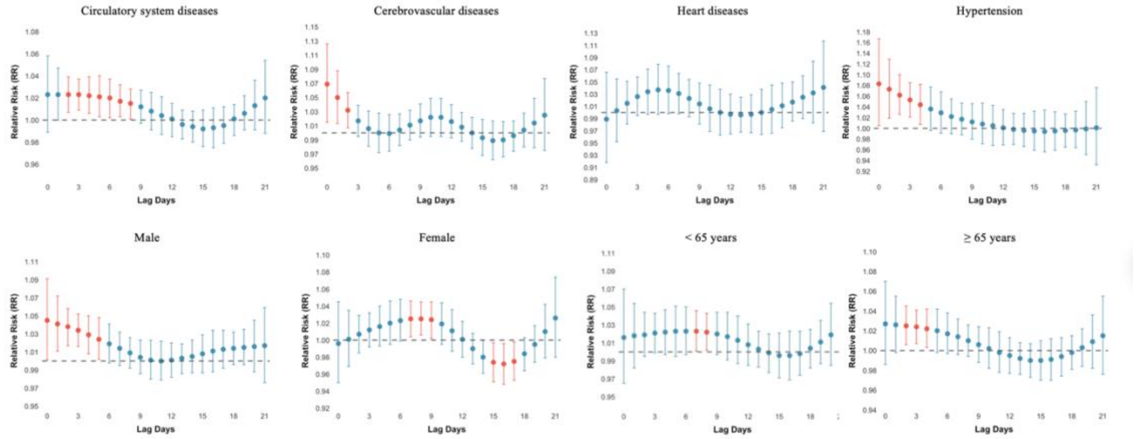


C.

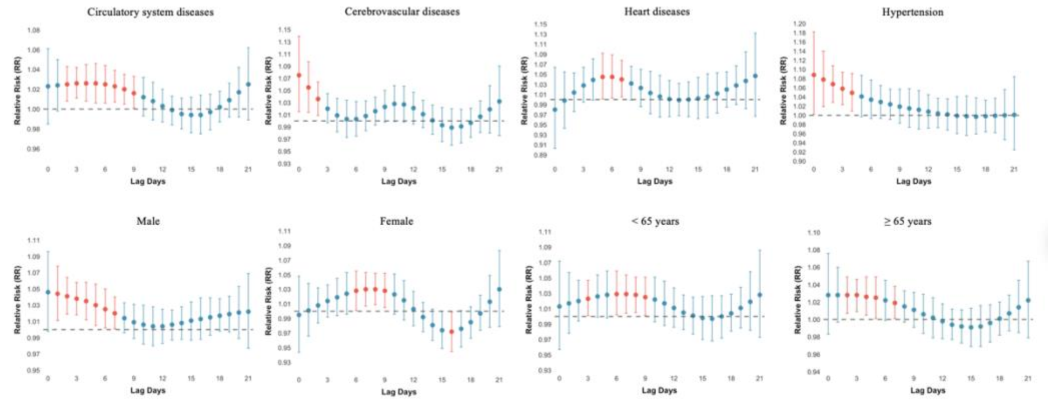

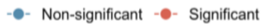


(A) T1；(B)T2；(C) T3

**Figure S3** The single-day lag effects of apparent low temperature on emergency call volume for Circulatory system diseases.

### 2.2.4. Cumulative Lag Effects of Low AT on EACs for Circulatory system diseases

The effects of low AT on the risk of EACs for circulatory system diseases exhibited pronounced cumulative lag patterns, with risks progressively increasing with sustained exposure to low AT. In the overall population, the cumulative lag effects were most prominent among males and individuals aged ≥65 years, persisting into the third week and reaching peak levels.

Among the different cold intensity categories, T3 showed the longest-lasting cumulative lag effects. In males, the cumulative effects emerged in the first week and continued to increase, peaking in the third week. Similarly, among individuals aged ≥65 years, cumulative effects began in the first week and persisted through the third week, reaching their maximum during this period. In contrast, no statistically significant cumulative lag effects were observed among females under any low AT conditions.

With respect to disease subtypes, hypertension and cerebrovascular diseases exhibited high sensitivity to all categories of low AT, with cumulative effects appearing early and persisting over extended lag periods, indicating a sustained adverse impact of low AT on these conditions. Notably, heart diseases did not show statistically significant cumulative lag effects under any low AT category (**Table S1**).

**Table S1** The cumulative lag effects of apparent low temperature on emergency call volume for circulatory system diseases

| | Circulatory system diseases | Cerebrovascular disease | Heart diseases | Hypertension | Male | Female | <65 years | ≥65 years |
|---|---|---|---|---|---|---|---|---|
| T1 | | | | | | | | |
| 0-7 | 1.160（1.062～1.267）* | 1.155（1.006～1.325）* | 1.164（0.957～1. 417） | 1.424（1.172～1. 730）* | 1.235（1.105～1.382）* | 1.088（0.960～1.233） | 1.157（1.011～1.324）* | 1.161（1.043～1.292）* |

| | | | | | | | | |
|---|---|---|---|---|---|---|---|---|
| 0-14 | 1.170（1.032～1.326）* | 1.238（1.020～1.502）* | 1.165（0.886～1.531） | 1.427（1.082～1.881）* | 1.235（1.054～1.448）* | 1.122（0.941～1.338） | 1.230（1.017～1.488）* | 1.131（0.973～1.316） |
| 0-21 | 1.175（0.999～1.382） | 1.233（0.960～1.584） | 1.310（0.914～1.853） | 1.377（0.963～1.970） | 1.328（1.082～1.631）* | 1.041（0.828～1.309） | 1.286（1.005～1.645）* | 1.107（0.910～1.346） |
| T2 | | | | | | | | |
| 0-7 | 1.193（1.082～1.314）* | 1.197（1.030～1.391）* | 1.189（0.959～1.473） | 1.491（1.205～1.846）* | 1.282（1.134～1.450）* | 1.109（0.967～1.271） | 1.183（1.021～1.371）* | 1.198（1.066～1.347）* |
| 0-14 | 1.234（1.075～1.417）* | 1.327（1.073～1.641）* | 1.241（0.919～1.675） | 1.566（1.154～2.125）* | 1.320（1.108～1.573）* | 1.170（0.964～1.421） | 1.292（1.047～1.594）* | 1.197（1.013～1.414）* |
| 0-21 | 1.264（1.055～1.513）* | 1.345（1.019～1.777）* | 1.419（0.958～2.100） | 1.536（1.030～2.290）* | 1.457（1.159～1.832）* | 1.098（0.851～1.416） | 1.360（1.033～1.791）* | 1.201（0.966～1.494） |
| T3 | | | | | | | | |
| 0-7 | 1.219（1.098～1.353）* | 1.232（1.048～1.447）* | 1.208（0.959～1.521） | 1.545（1.228～1.944）* | 1.320（1.156～1.506）* | 1.125（0.971～1.304） | 1.203（1.026～1.411）* | 1.228（1.082～1.392）* |
| 0-14 | 1.289（1.111～1.496）* | 1.403（1.116～1.764）* | 1.306（0.946～1.804） | 1.687（1.213～2.346）* | 1.393（1.153～1.682）* | 1.211（0.982～1.493） | 1.343（1.070～1.685）* | 1.253（1.047～1.449）* |
| 0-21 | 1.340（1.102～1.630）* | 1.444（1.068～1.952）* | 1.521（0.995～2.327） | 1.678（1.087～2.590）* | 1.570（1.224～2.013）* | 1.147（0.870～1.512） | 1.422（1.055～1.917）* | 1.285（1.015～1.628）* |

## Sensitivity Analyses

**Table S2** Sensitivity analyses for the association between apparent temperature (AT) and EACs

| Analysis type | T1 (95% CI) | T2 (95% CI) | T3 (95% CI) |
|---|---|---|---|
| Main model | 1.175(0.999-1.382) | 1.264(1.055-1.513) | 1.340(1.102-1.630) |
| lag: 0-14 | 1.160 (0.985–1.366) | 1.245 (1.042–1.488) | 1.295(1.065-1.575) |
| lag: 0-28 | 1.190 (1.010–1.402) | 1.285 (1.075–1.535) | 1.362(1.118-1.658) |
| AT definition: T1=P10, T2=P5, T3=P1 | 1.175(0.999-1.382) | 1.272 (1.060–1.526) | 1.365(1.115-1.672) |
| AT definition: T1=P10, T2=P2.5, T3=P1 | 1.150 (0.972–1.360) | 1.238 (1.030–1.490) | 1.325(1.085-1.617) |
| df for time 6 | 1.165 (0.990–1.372) | 1.255 (1.048–1.502) | 1.312(1.078-1.596) |
| df for time 8 | 1.185 (1.005–1.395) | 1.275 (1.065–1.525) | 1.355(1.110-1.653) |
| df for RH 4 | 1.170 (0.995–1.378) | 1.260 (1.052–1.508) | 1.348(1.105-1.642) |
| df for RH 5 | 1.155 (0.980–1.360) | 1.248 (1.040–1.495) | 1.330(1.090-1.623) |

**Table S3** Winter-only sensitivity analyses of additive interactions (RERI) between apparent low temperature and $PM_{2.5}$ joint exposure.

| Outcome | Model | RERI(P1T3) | RERI(P2T2) |
|---|---|---|---|
| Circulatory system | Annual | 0.085(-0.037-0.206) | -0.089(-0.153- -0.024) |
| diseases | Winter only | 0.226(-0.107-0.559) | 0.146(-0.075-0.367) |
| Cerebrovascular disease | Annual | 0.073(-0.114-0.260) | -0.085(-0.183-0.014) |
| | Winter only | 0.111(-0.431-0.654) | 0.077(-0.253-0.406) |
| Heart diseases | Annual | 0.103(-0.154-0.360) | -0.031(-0.167-0.104) |
| | Winter only | 0.546(0.070-1.022) | 0.294(-0.125-0.713) |

| | | | |
|---|---|---|---|
| Hypertension | Annual | 0.322(0.061-0.583) | -0.282(-0.446- -0.118) |
| | Winter only | 0.620(-0.145-1.384) | 0.487(0.051-0.922) |

**Table S4** Comparison of AIC values for different models across various maximum lag days

| | lag | AIC |
|---|---|---|
| Single-exposure AT model（T3） | 14 | 23353.84 |
| | 21 | 23314.36 |
| | 28 | 23326.15 |
| Joint exposure model (P1T3) | 14 | 22946.01 |
| | 21 | 22948.13 |
| | 28 | 22949.41 |

**Table S5** The results of AIC of combined events definitions

| Combined events | Circulatory system diseases |
|---|---|
| P1T1 | 22939.4 |
| P1T2 | 22946.0 |
| P1T3 | 22898.4 |
| P2T1 | 22950.1 |
| P2T2 | 22928.9 |
| P2T3 | 22940.8 |
| P3T1 | 22944.8 |
| P3T2 | 22951.5. |
| P3T3 | 22944.8 |
| P4T1 | 22950.2 |
| P4T2 | 22942.6 |

A.

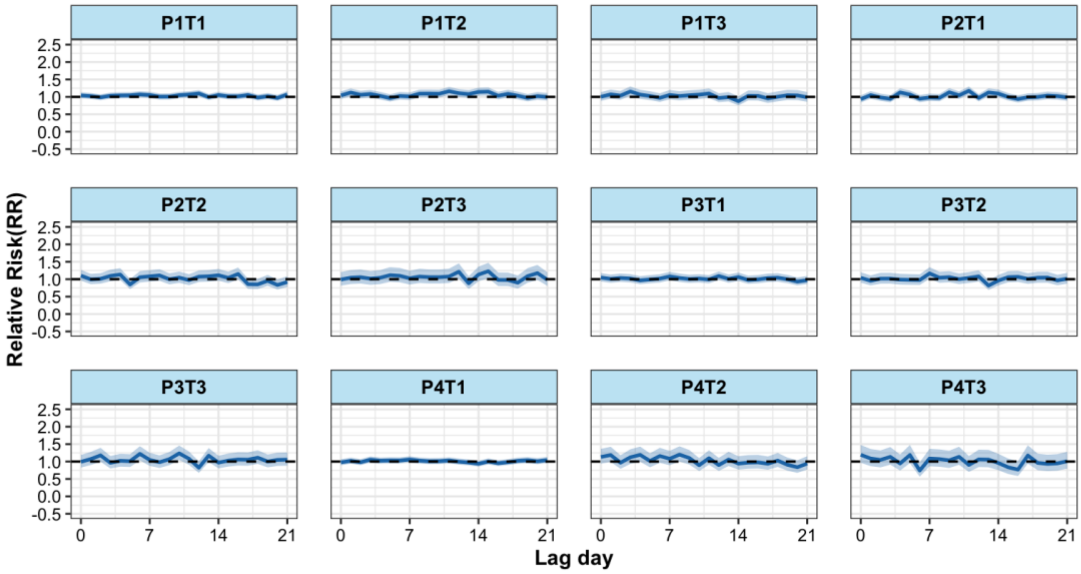

P1T1
P1T2
P1T3
P2T1
P2T2
P2T3
P3T1
P3T2
P3T3
P4T1
P4T2
P4T3
Relative Risk(RR)
Lag day
2.5
2.0
1.5
1.0
0.5
0.0
-0.5
0
7
14
21


B.

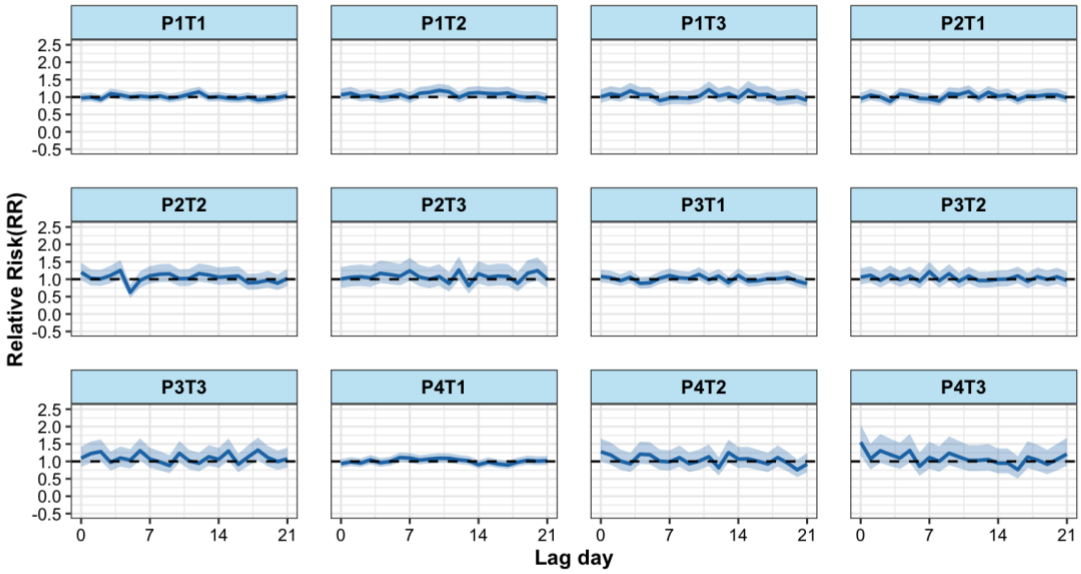

P1T1
P1T2
P1T3
P2T1
P2T2
P2T3
P3T1
P3T2
P3T3
P4T1
P4T2
P4T3
Relative Risk(RR)
Lag day
2.5
2.0
1.5
1.0
0.5
0.0
-0.5
0
7
14
21


C.

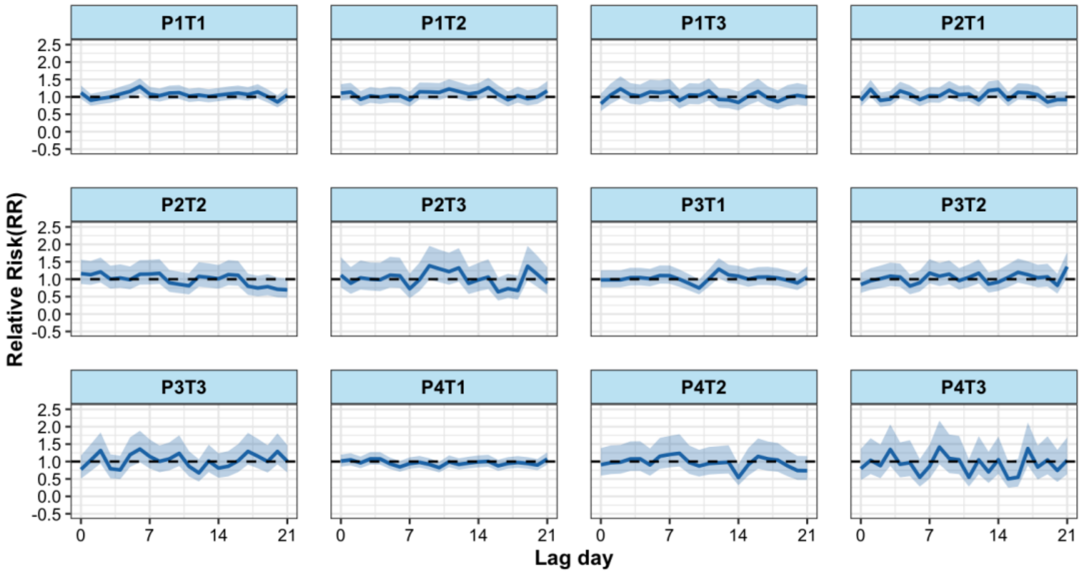
P1T1
P1T2
P1T3
P2T1
P2T2
P2T3
P3T1
P3T2
P3T3
P4T1
P4T2
P4T3
Relative Risk(RR)
Lag day

D.

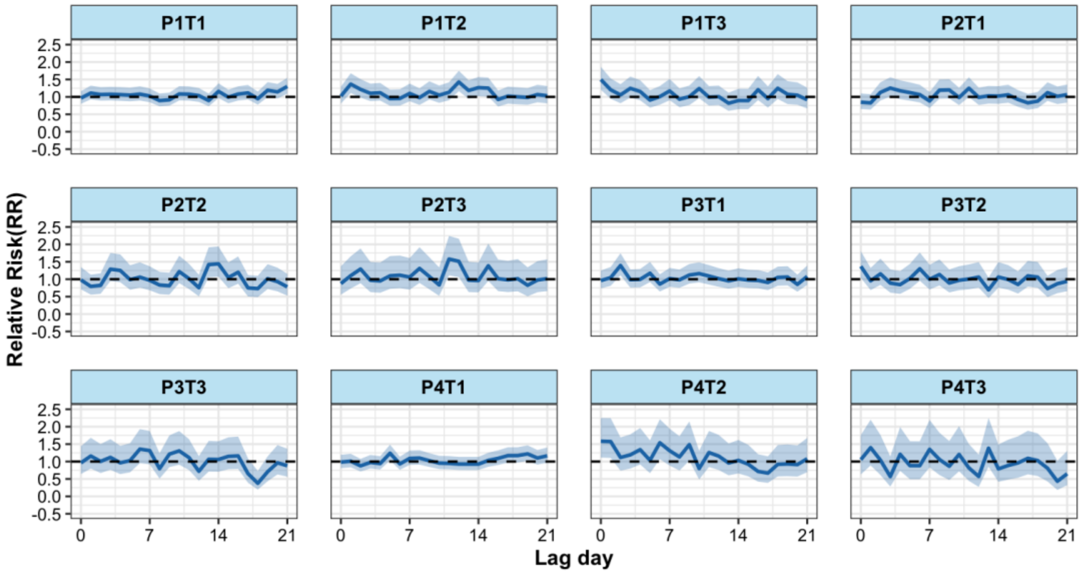
P1T1
P1T2
P1T3
P2T1
P2T2
P2T3
P3T1
P3T2
P3T3
P4T1
P4T2
P4T3
Relative Risk(RR)
Lag day

E.

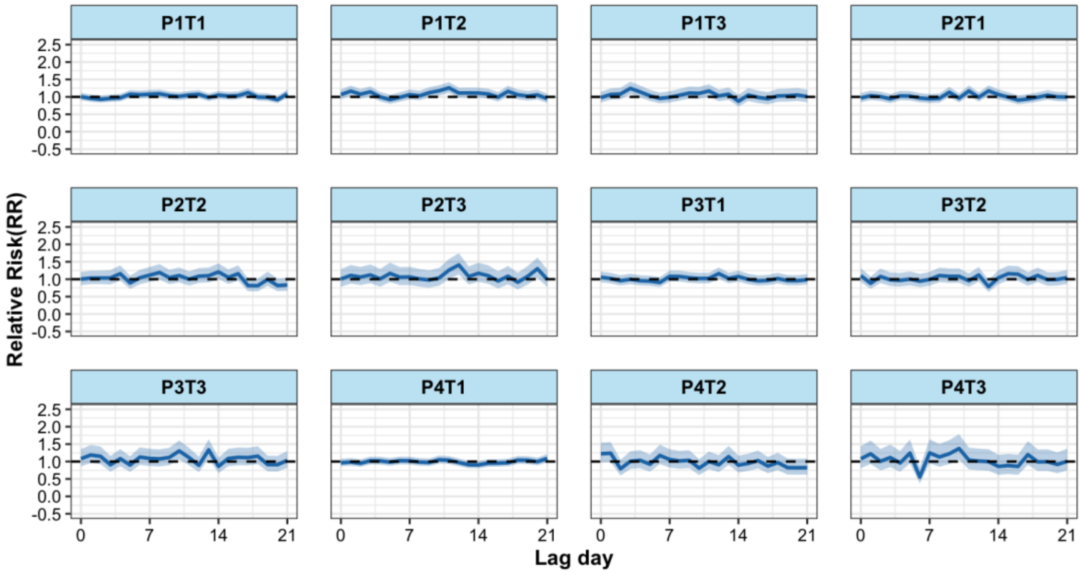
P1T1
P1T2
P1T3
P2T1
P2T2
P2T3
P3T1
P3T2
P3T3
P4T1
P4T2
P4T3
Relative Risk(RR)
Lag day

F.

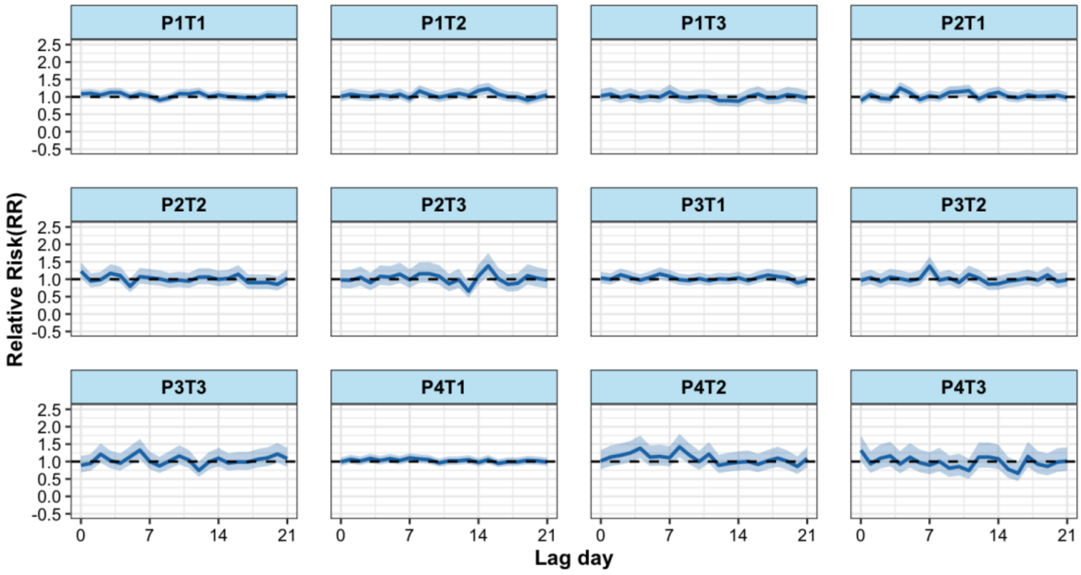
P1T1
P1T2
P1T3
P2T1
P2T2
P2T3
P3T1
P3T2
P3T3
P4T1
P4T2
P4T3
Relative Risk(RR)
Lag day

G.

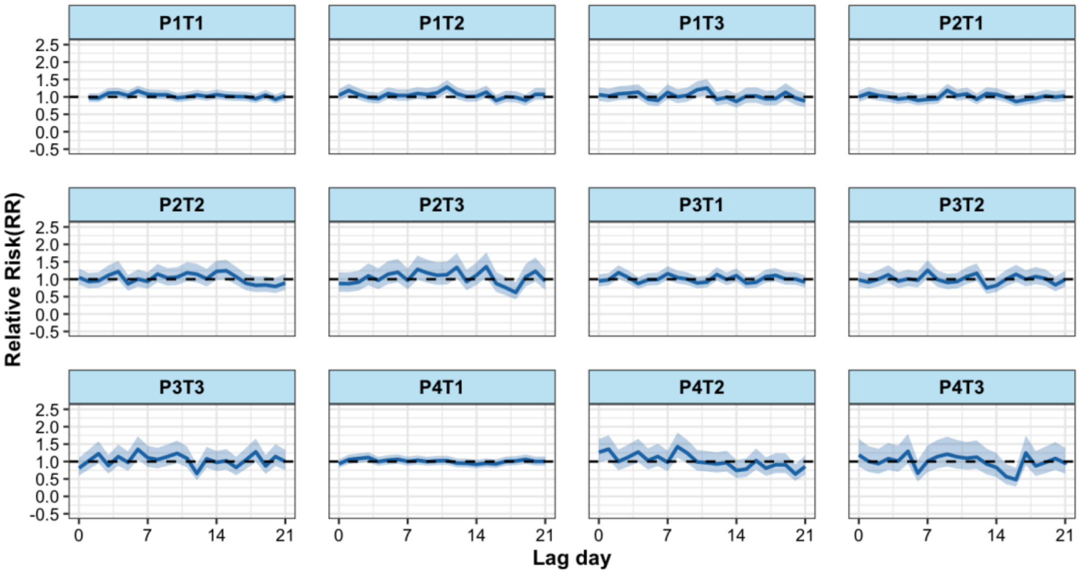


H.

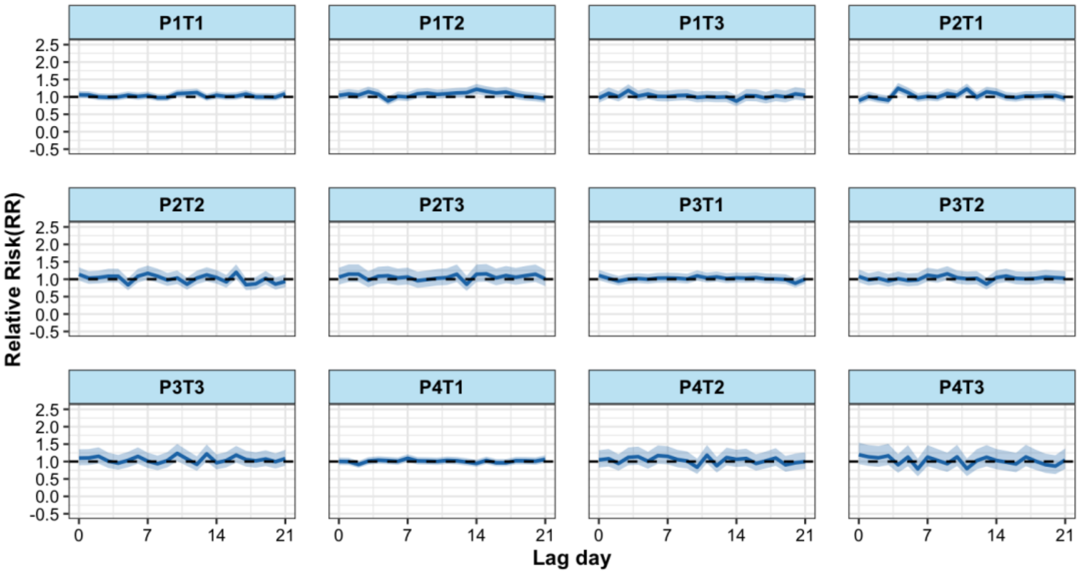


(A)circulatory system diseases; (B) cerebrovascular diseases; (C) heart diseases; (D) hypertension; (E) males; (F) females; (G) <65 years; (H) ≥65 years.

**Figure S4** Extended lag-response curves for joint exposure categories (lag 0–21 days)

Compared with the primary lag 0–14 day analyses presented in Figure 1, similar temporal patterns were observed during the early lag period. However, progressively wider confidence intervals and increased fluctuations in effect estimates were observed beyond lag 14 days, indicating reduced model stability under extended lag specifications.
